# The Nitrated Fatty Acid 10-Nitro-oleate Diminishes Severity of LPS-Induced Acute Lung Injury in Mice

**DOI:** 10.1155/2012/617063

**Published:** 2012-07-26

**Authors:** Aravind T. Reddy, Sowmya P. Lakshmi, Raju C. Reddy

**Affiliations:** Department of Medicine, Division of Pulmonary, Allergy and Critical Care Medicine, Emory University and Atlanta VA Medical Center, Atlanta, GA 30033, USA

## Abstract

Acute lung injury (ALI) is an inflammatory condition culminating in respiratory failure. There is currently no effective pharmacological treatment. Nitrated fatty acids (NFAs) have been shown to exert anti-inflammatory effects. We therefore hypothesized that delivery of NFAs directly to the site of inflammation would reduce the severity of ALI. Pulmonary delivery of 10-nitro-oleate following endotoxin-induced ALI in mice reduced markers of lung inflammation and injury, including capillary leakage, lung edema, infiltration of neutrophils into the lung, and oxidant stress, as well as plasma levels of proinflammatory cytokines. Nitro-oleate delivery likewise downregulated expression of proinflammatory genes by alveolar macrophages, key cells in regulation of lung inflammation. These effects may be accounted for by the observed increases in the activity of PPAR-**γ** and the PPAR-**γ**-induced antioxidant transcription factor Nrf2, together with the decreased activity of NF-**κ**B. Our results demonstrate that pulmonary delivery of NFAs reduces severity of acute lung injury and suggest potential utility of these molecules in other inflammatory lung diseases.

## 1. Introduction

A variety of pulmonary and extrapulmonary insults can result in acute lung injury (ALI), which is characterized by capillary leakage and resulting pulmonary edema and hypoxemia [1]. These multiple origins of ALI are reflected in different animal models of the disease, of which pulmonary administration of bacterial endotoxin (lipopolysaccharide; LPS) is among the most common. Regardless of the precipitating cause, the earliest phases of ALI feature severe neutrophil-rich alveolar inflammation [2] and associated oxidant stress [3] that represent the proximate causes of much or all of the subsequent pulmonary injury. ALI morbidity and mortality remain high and there is no effective pharmacotherapy [4], underlining the urgent need for improved treatment modalities.

Peroxisome proliferator-activated receptor *γ* (PPAR-*γ*) plays a central role in many of the feedback loops that normally limit inflammation and lead to its resolution [5–9] and is therefore a promising target for ALI pharmacotherapy. Among the many anti-inflammatory effects attributable to PPAR-*γ* activation are diminished increases in reactive oxygen species, cytokines, chemokines, and adhesion molecules [10]. A major mechanism underlying these actions is decreased activity of proinflammatory transcription factors such as NF-*κ*B, AP-1, and STAT [5, 6]. Synthetic PPAR-*γ* agonists are widely used for treatment of diabetes but have been associated with adverse effects. A wide variety of endogenous molecules are known to activate PPAR-*γ*, but most either exhibit low potency or are present at low concentrations, leading to uncertainty regarding their physiological role. 

Nitrated fatty acids (NFAs) are activating ligands for all three PPARs, exhibiting their greatest potency as PPAR-*γ* agonists [11, 12]. They have also been shown to exhibit a number of PPAR-*γ*-dependent effects, including induction of adipogenesis [11] and of CD36 receptor expression by macrophages [12]. NFAs are produced endogenously by nonenzymatic reaction of NO or its inorganic reaction products with naturally present unsaturated fatty acids [13], with positional isomers of nitro-oleic acid (OA-NO_2_) and nitrolinoleic acid (LNO_2_) found at the highest concentrations in human plasma [11, 14]. The suggested PPAR-*γ*-mediated anti-inflammatory effects of NFAs have been supported by *in vitro* studies [15, 16], but *in vivo* and potentially translational studies of such effects have been limited. These considerations led us to investigate the ability of treatment with OA-NO_2_, the most potent PPAR-*γ*-activating NFA [11], to reduce the severity of inflammation and lung injury in a murine model of ALI induced by LPS. In order to maximize pulmonary availability, OA-NO_2_ was delivered directly to the lung via the intratracheal route.

## 2. Materials and Methods

### 2.1. Animals

Male C57BL/6 mice were obtained from Jackson Laboratories (Bar Harbor, ME) and were used at 6–8 weeks of age (20–25 g). All studies were performed according to protocols reviewed and approved by the Atlanta Veterans Affairs Medical Center Institutional Animal Care and Use Committee. 

### 2.2. OA-NO_2_ and LPS Administration

Mice were anesthetized with 90 mg/kg ketamine and 10 mg/kg xylazine, administered via intraperitoneal injection, and a tracheotomy was performed. ALI was then induced by intratracheal (i.t.) injection of 50 *μ*g of endotoxin (LPS) prepared from *Escherichia coli* O111:B6 (Sigma-Aldrich, St. Louis, MO). Thirty min later mice were injected i.t. with 50 *μ*g OA-NO_2_ (Cayman Chemical, Ann Arbor, MI) in 50 *μ*L of 10% DMSO or with vehicle. After a further 5.5 h the mice were euthanized, plasma was obtained, and lung and BAL fluid samples were collected. Neutrophil infiltration into the lung peaks approximately 6 h after i.t. LPS administration [17].

### 2.3. Lung Wet:Dry Weight Ratio

As an index of lung edema, the amount of extravascular lung water was calculated. The lower lobe of the right lung was ligated and excised and the wet weight was recorded. The lung was then placed in an incubator at 60°C for 24 h to obtain the dry weight. The wet:dry ratio was calculated by dividing the wet weight by the dry weight.

### 2.4. Bronchoalveolar Lavage (BAL) Fluid Collection and Cell Count

Following removal of the lung's lower right lobe, BAL fluid was collected by flushing 3 × 1 mL of phosphate-buffered saline (PBS) containing 0.1 mM EDTA into the lung via a tracheal cannula. The pooled BAL fluid was centrifuged at 500 ×*g* at 4°C for 5 min. Pelleted cells were then resuspended in 1 mL of PBS. Total cell number was counted by hemocytometer and a differential cell count was performed by cytospin staining with Diff-Quik (Siemens, Newark, DE).

### 2.5. BAL Fluid Protein

Increase in BAL fluid protein concentration was taken as a measure of increased permeability of alveolar-capillary barriers. Total protein concentration in the supernatant following BAL fluid centrifugation was determined using the BCA Protein Assay kit (Pierce, Rockford, IL). 

### 2.6. Lung Histopathology

The lungs were inflated and fixed with 10% neutral formalin overnight at room temperature. Lung tissue was dehydrated with increasing ethanol (EtOH) concentrations and then embedded in paraffin. Five-micrometer-thick paraffin sections were stained with hematoxylin and eosin (H&E). 

### 2.7. Assessment of Capillary Leakage

To further assess lung permeability, 50 mg/kg of Evans Blue Dye (EBD; Sigma-Aldrich, St. Louis, MO) dissolved in 200 *μ*L of PBS was injected into the tail veins of mice following ALI induction. After 30 min, the animals were euthanized and the lungs perfused with 5 mL PBS, after which the lungs were excised *en bloc* and snap-frozen in liquid nitrogen. The frozen lungs were then homogenized in 2 mL PBS. The homogenate was diluted with 2 vol of formamide and incubated at 60°C for 18 h, followed by centrifugation at 5,000 ×*g* for 30 min. The supernatant was collected and absorbance was measured at 620 and 740 nm. The EBD concentration was determined from standard absorbance curves evaluated in parallel. Correction for contaminating heme pigments was calculated by the formula E_620_(EBD) = E_620_ − (1.426 × E_740_ + 0.030). The EBD concentration was expressed as *μ*g per g of lung.

### 2.8. Measurement of Myeloperoxidase Activity

As an index of neutrophil infiltration, BAL fluid and tissue-associated myeloperoxidase (MPO) activity was determined. Frozen lung tissues were thawed, weighed, homogenized, and sonicated on ice in radioimmunoprecipitation assay buffer (RIPA). After centrifugation at 10,000 ×*g* at 4°C for 20 min, the supernatant was collected and used for determination of MPO activity by a commercially available fluorometric assay kit (700160; Cayman Chemical) according to the manufacturer's instructions. Results were expressed as nmol/min-ml. Similar measurements were performed on BAL fluid supernatant.

### 2.9. Measurement of Oxidant Stress

Hydrogen peroxide (H_2_O_2_) production in lung tissue was determined using the Amplex Red Hydrogen Peroxide Assay kit (Molecular Probes, Eugene, OR) according to the manufacturer's directions. The concentrations of nitrate and malondialdehyde (MDA) in lung homogenates were measured using commercially available colorimetric assay kits (Cayman Chemical) according to the manufacturer's instructions. 

### 2.10. Measurement of Plasma Cytokine Levels

Plasma levels of tumor necrosis factor-*α* (TNF-*α*), interleukin-6 (IL-6), keratinocyte chemoattractant (KC), and macrophage inflammatory protein-2 (MIP-2) were measured using enzyme-linked immunosorbent assay (ELISA) kits (R&D Systems, Minneapolis, MN) according to the manufacturer's instructions. 

### 2.11. RNA Isolation and Quantitative Real-Time RT-PCR

Cells pelleted from BAL fluid were resuspended in DMEM supplemented with 10% fetal bovine serum, allowed to adhere to tissue culture-treated six-well plates for 1 h, and then washed twice to remove nonadherent cells. Adherent alveolar macrophages were lysed, and RNA was isolated using RNeasy Mini kit (Qiagen, Valencia, CA), with cDNA being generated from 100 ng of total RNA using MultiScribe reverse transcriptase (Applied Biosystems, Foster City, CA) employing random and oligo dT primers. Real-time quantitative PCR was performed using 100 ng cDNA with 2X SYBR Green Master mix (Applied Biosystems) and specific primers for the genes of interest (Table 1). These experiments were performed on an AB 7500 fast thermal cycler using a three-step protocol employing the melting curve method. The average of each gene cycle threshold (*C*_*t*_) was determined for each experiment. Relative cDNA levels (2^−ΔΔCt^) for the genes of interest were determined by using the comparative *C*_*t*_ method, which generates the ΔΔCt as the difference between the gene of interest and the housekeeping genes glyceraldehyde-3-phosphate dehydrogenase (GAPDH) and 9s rRNA for each sample. Each averaged experimental gene expression sample was compared to the averaged control sample, which was set to 1.

### 2.12. Transient Transfection Assay

PPAR-*γ* activity in A549 cells (ATCC, Rockville, MD) was determined as previously described [18]. Briefly, cells were transiently co-transfected either with a plasmid containing the luciferase gene under regulation by four Gal4 DNA-binding elements (UAS_G_× 4 TK-luciferase) and a plasmid containing the PPAR-*γ* ligand-binding domain fused to the Gal4 DNA-binding domain or with the luciferase gene under control of the peroxisome proliferator response element (PPRE) isolated from the fatty acid transport protein. All transfections were performed using Lipofectamine 2000 (Invitrogen) according to the manufacturer's instructions. Following treatment with test compounds, activation was measured using the Dual-Luciferase Reporter Assay System (Promega, Madison, WI) according to the manufacturer's instructions. 

### 2.13. Transcription Factor DNA-Binding Activity Assay

Nuclear proteins were extracted using a nuclear extraction kit (Active Motif, Carlsbad, CA) according to the manufacturer's protocol. The protein concentration was determined using the BCA Protein Assay kit (Pierce). Nuclear extracts were used to quantify DNA-binding activity of PPAR-*γ*, nuclear factor (erythroid-derived 2)-like 2 (Nrf2), and the p65 subunit of NF-*κ*B using ELISA-based TransAM kits (40096, 40696, and 50296; Active Motif) according to the manufacturer's instructions.

### 2.14. Molecular Modeling and Computer Simulations of Binding of OA-NO_2_ with PPAR-*γ*

*In silico* construction of OA-NO_2_ was carried out using Chem Office (ChemDraw and Chem3D; CambridgeSoft Corp., Cambridge, MA). To avoid steric hindrance and clashes that can appear in the building process, the models obtained were subjected to geometry optimization using GaussView with a protocol of 300 steps of conjugate gradients. Each model then was optimized using a semi-empirical method such as PM3 as implemented in the Gaussian98 package of programs.

Autodock 4.2 (with Lamarckian Genetic Algorithm) or ArgusLab 4 was used to generate the starting complexes. An elitism value of 1 was used, together with probabilities of mutation and crossing-over of 0.03 and 0.07, respectively. From the best solutions obtained according to these parameters, those defined by the user as exhibiting the best probabilities—in our case, 0.07—were further refined by a local search method. Autodock defines the conformational space implementing grids over the entire space of possible solutions. With the aim of testing the ability of Autodock to converge into solutions that are inside the PPAR-*γ* ligand-binding domain, a grid of 70 Å per side with 0.4 Å spacing between points was set up in such a way that it covered both the external surface and the internal cavity of the PPAR-*γ* ligand-binding domain. The following procedure was employed for the OA-NO_2_-PPAR-*γ* ligand-binding domain docking simulations: 150 runs were done for each OA-NO_2_-PPAR-*γ* ligand-binding domain. At the end of each run, the complexes were separated into clusters according to their lowest root mean square deviation (RMSD), and the best score value based on a free empiric energy function was determined. Cluster complexes whose average score was −11.50 kcal-mol/L with respect to the best energy obtained in that run were selected. The selected final complexes were optimized using the semi-empirical PM3 method as a refining procedure with Gaussian98.

Constant-volume, constant-temperature molecular dynamics (MD) simulations of the complexes were performed on the Discover, version 2.7 (Biosym Technologies, Inc., San Diego, CA) and MD simulation programs in Chem Office. Energy minimizations were conducted for two systems, both of which assumed 1 molecule of OA-NO_2_, 1 molecule of PPAR-*γ*, and water molecules. The systems simulated were; (i) OA-NO_2_ and PPAR-*γ* separated from each other by a distance greater than the non-bonded cutoff distance (>8.5 Å); (ii) OA-NO_2_ complexed with the PPAR-*γ* ligand-binding domain. Each molecular system was contained in a box size of 25.0 × 25.0 × 37.0 Å with periodic boundary conditions. The step size was 2 femtoseconds (fsec). To start the simulations, different seed numbers were used for initial Maxwellian velocity distribution for each system. Simulations were continued and the coordinates were saved for analysis every 2 fsec.

### 2.15. Statistical Analysis

Data are presented as mean ± SD. Differences between groups were analyzed using ANOVA, followed by a Bonferroni multiple comparison test using GraphPad Prism 5.03 (GraphPad Software, La Jolla, CA). *P* < 0.05 was considered significant.

## 3. Results

### 3.1. *In Silico* Binding of OA-NO_2_ to PPAR-*γ*

**Figure 1:**
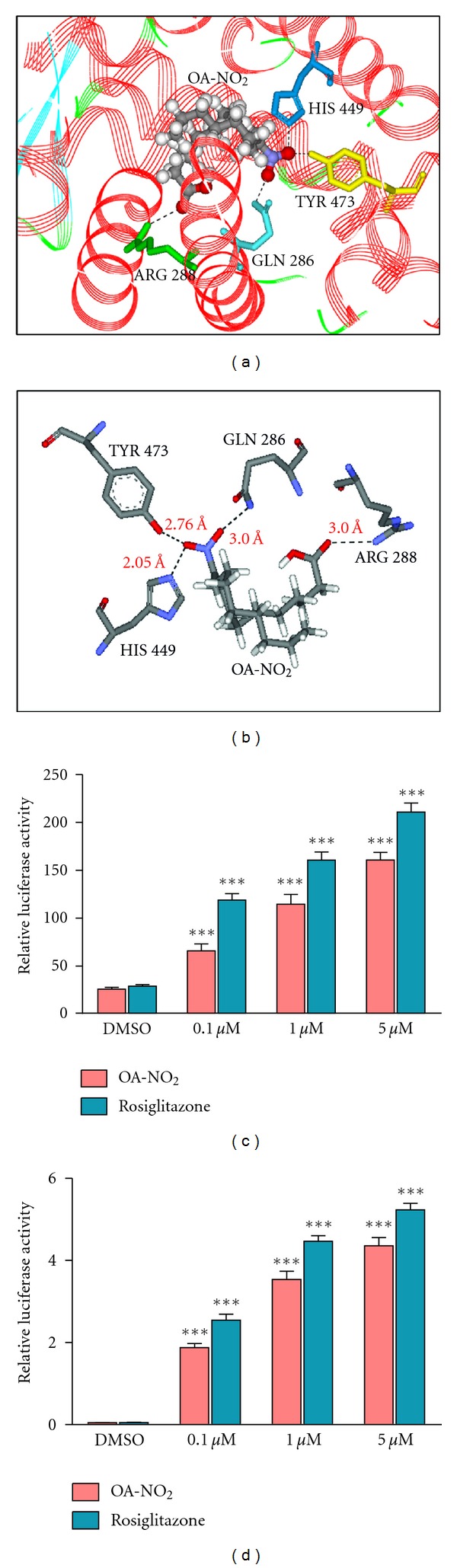
OA-NO_2_ activates PPAR-*γ* in airway epithelial cells. Binding of OA-NO_2_ to the PPAR-*γ* ligand-binding domain was modeled *in silico*. Results are presented as (a) space-filling representation; (b) schematic representation showing interactions of PPAR-*γ* residues with specific groups in the OA-NO_2_ ligand. Hydrogen bonds are indicated by dotted lines. A549 cells were transiently transfected with pRLSV40 and with one of the following constructs: (c) PPAR-dependent luciferase reporter, pFATPluc; (d) PPAR-*γ* GAL4 reporter system (UAS_G_× 4 TK-luciferase + GAL4-PPAR-*γ*). Cells were then incubated with vehicle (DMSO), OA-NO_2_ (0.1–5 *μ*M), or rosiglitazone (0.1–5 *μ*M). After 24 h, luciferase activity was measured with a dual luciferase activity kit and normalized to that of *Renilla* luciferase. Data are representative of one of two independent experiments; *n* = 6; ***P < 0.001 versus vehicle.

Although the crystal structure of LNO_2_ bound to the PPAR-*γ* ligand binding site has been reported [19], no similar information is available for OA-NO_2_. We accordingly used *in silico* methods to determine the likely binding mode for this compound. As shown in Figures 1(a) and 1(b), OA-NO_2_ is well accommodated within the PPAR-*γ* ligand-binding site, with best pose energy of −11.50 kcal/mol, and exhibits the appropriate interactions and hydrogen bonds with the PPAR-*γ* amino acid residues (ARG 288, GLN 286, HIS 449, and TYR 473) that are known to be important for PPAR-*γ* activation.

### 3.2. OA-NO_2_ Activates PPAR-*γ* *In Vitro*

To confirm the ability of NFAs to activate PPAR-*γ* we utilized the GAL4 reporter system in A549 airway epithelial cells. Transfected cells were treated with 0.1, 1, or 5 *μ*M concentrations of OA-NO_2_ or, as a positive control, of the synthetic PPAR-*γ* agonist rosiglitazone. Each compound demonstrated dose-dependent activation as a result of binding to the construct's PPAR-*γ* ligand-binding domain, with similar activation for given molar concentrations (Figure 1(c)). To demonstrate activation of endogenous PPAR-*γ*, A549 cells were similarly transfected with the luciferase gene under control of a PPAR response element. This assay likewise demonstrated dose-dependent activation by both OA-NO_2_ and rosiglitazone (Figure 1(d)). 

### 3.3. Pulmonary Administration of OA-NO_2_ Diminishes Severity of LPS-Induced Lung Inflammation

Pulmonary inflammation is a crucial feature of ALI. Since the nuclear receptor PPAR-*γ* is known to exert a variety of anti-inflammatory effects and unsaturated long-chain NFAs are activating ligands for PPAR-*γ*, we hypothesized that pulmonary delivery of an NFA would diminish the severity of ALI. To test this hypothesis, we utilized a well-established murine model of ALI induced by i.t. administration of 50 *μ*g of LPS. Thirty min after LPS injection, 50 *μ*g of OA-NO_2_ in 10% DMSO was delivered to the lungs via the i.t. route. Control mice received vehicle without NFA. After a further 5.5 h the mice were euthanized, the lower right lobe excised for assessment of edema by wet:dry weight ratio, BAL fluid collected, and the lungs excised for histopathological examination and measurement of inflammation-association molecular markers. Plasma was obtained at the same time.

**Figure 2:**
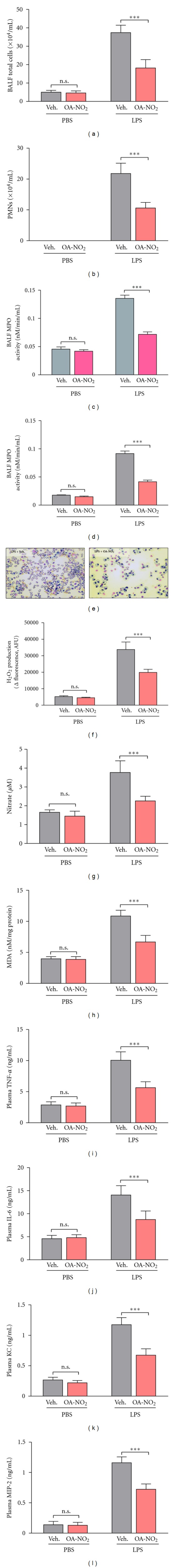
Pulmonary delivery of OA-NO_2_ reduces LPS-induced lung inflammation. Induction of ALI by i.t. injection of LPS (50 *μ*g) was followed 30 min later by i.t. administration (50 *μ*L) of OA-NO_2_ (50 *μ*g) or vehicle (10% DMSO). After a further 5.5 h, BAL fluid, plasma, and lung samples were obtained. (a) Total cell and (b) neutrophil number in BAL fluid. Myeloperoxidase activity in (c) lung tissue and (d) BAL fluid. (e) Microscopic examination following staining of BAL fluid. (f) H_2_O_2_ production, (g) nitrate concentration, and (h) malonaldehyde/protein ratio in lung. Plasma levels of (i) TNF-*α*, (j) IL-6, (k) KC, and (l) MIP-2. Data are representative of one of two independent experiments with *n* = 6–8 mice per group; ***P < 0.001.

A prominent aspect of pulmonary inflammation is infiltration of neutrophils (polymorphonuclear leukocytes; PMNs) into the lungs and thus into BAL fluid. We observed an LPS-induced increase in the total number of cells in BAL fluid (Figure 2(a)), and differential staining indicated that neutrophils accounted for most of the increase (Figure 2(b)). Both increases were significantly attenuated by OA-NO_2_ treatment. Similar results were seen for measurements of myeloperoxidase in both lung (Figure 2(c)) and BAL fluid (Figure 2(d)). MPO is found in the pulmonary system in association with PMNs and is thus a marker for their presence. These results were confirmed by direct microscopic examination of BAL fluid (Figure 2(e)).

Inflammation is also characterized by oxidant stress, which directly injures lung tissues. Oxidant stress reflects production of reactive oxygen species such as H_2_O_2_ and superoxide, predominantly by macrophages and neutrophils, and of the reactive nitrogen species NO by a variety of cell types. As expected, measurement of H_2_O_2_ demonstrated a large increase following LPS administration that was reduced by OA-NO_2_ treatment (Figure 2(f)). Similar results were seen for nitrate, the end product of NO metabolism (Figure 2(g)). Levels of the lipid oxidation product MDA that provides an index of overall oxidative stress, and MDA levels followed the same pattern as the other two markers of oxidant generation (Figure 2(h)).

Inflammation in ALI is likewise associated with release of proinflammatory cytokines and chemokines by neutrophils, macrophages, and other cells. We measured the proinflammatory cytokines TNF-*α* (Figure 2(i)) and IL-6 (Figure 2(j)), the chemokine KC, which activates and attracts neutrophils (Figure 2(k)), and the related chemokine MIP-2, which has effects similar to KC (Figure 2(l)). Plasma levels of all four markers were greatly increased by LPS administration, but this increase was significantly reduced by OA-NO_2_ treatment.

### 3.4. Pulmonary Administration of OA-NO_2_ Diminishes Capillary Permeability and Severity of LPS-Induced Lung Injury

**Figure 3:**
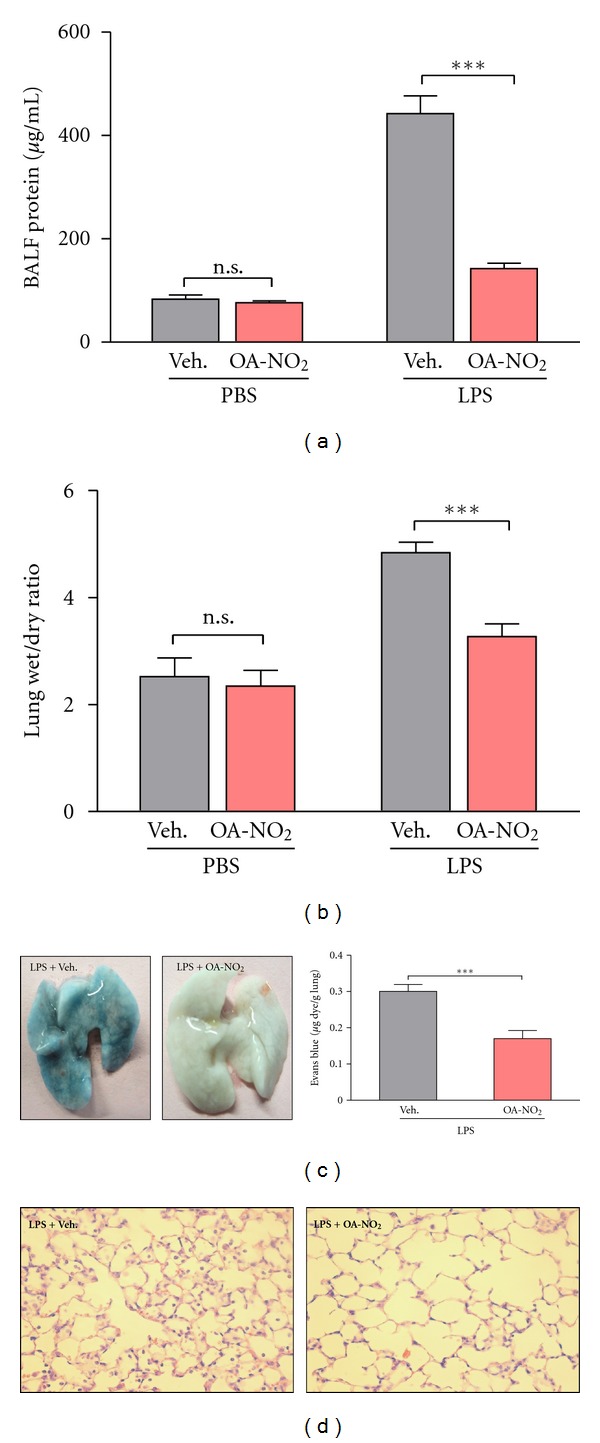
Pulmonary delivery of OA-NO_2_ reduces LPS-induced lung injury. Induction of ALI by i.t. injection of LPS (50 *μ*g) was followed 30 min later by i.t. administration (50 *μ*L) of OA-NO_2_ (50 *μ*g) or vehicle (10% DMSO). After a further 5.5 h, BAL fluid and lung samples were obtained. (a) Protein concentration in BAL fluid. (b) Ratio of lung wet:dry weight. (c) Extravasation of Evans Blue dye into the lung following intravenous injection was photographed and quantitated by spectrophotometry. (d) The lung was examined histologically following H&E staining. Data are representative of one of two independent experiments with *n* = 6–8 mice per group; ***P < 0.001.

Increased capillary permeability results in lung edema, a driving force for the hypoxemia that is observed in ALI. It also allows escape of plasma proteins into the alveolar space, which can then be detected in BAL fluid. We find that OA-NO_2_ treatment attenuates the LPS-induced increase in BAL fluid protein concentration (Figure 3(a)). The increased vascular permeability following LPS administration allowed EBD to extravasate from the vasculature into the lung parenchyma, turning these lungs deep blue (~0.3 *μ*g dye per g lung). This extravasation of EBD was significantly reduced by OA-NO_2_ treatment, however, resulting in only a pale blue appearance of the lung (~0.17 *μ*g dye per g lung; Figure 3(c)). The lung edema that results from capillary permeability is reflected in the wet:dry weight ratio, and LPS-induced increases in this parameter were also reduced by OA-NO_2_ treatment (Figure 3(b)). Direct histopathological examination of the lung following H&E staining further confirmed significant lung inflammation and injury in mice treated with vehicle but much less severe abnormalities in those that had received OA-NO_2_ (Figure 3(d)).

### 3.5. OA-NO_2_ Produces Anti-Inflammatory Alterations in Activity of PPAR-*γ*, NF-*κ*B, and Nrf2

**Figure 4:**
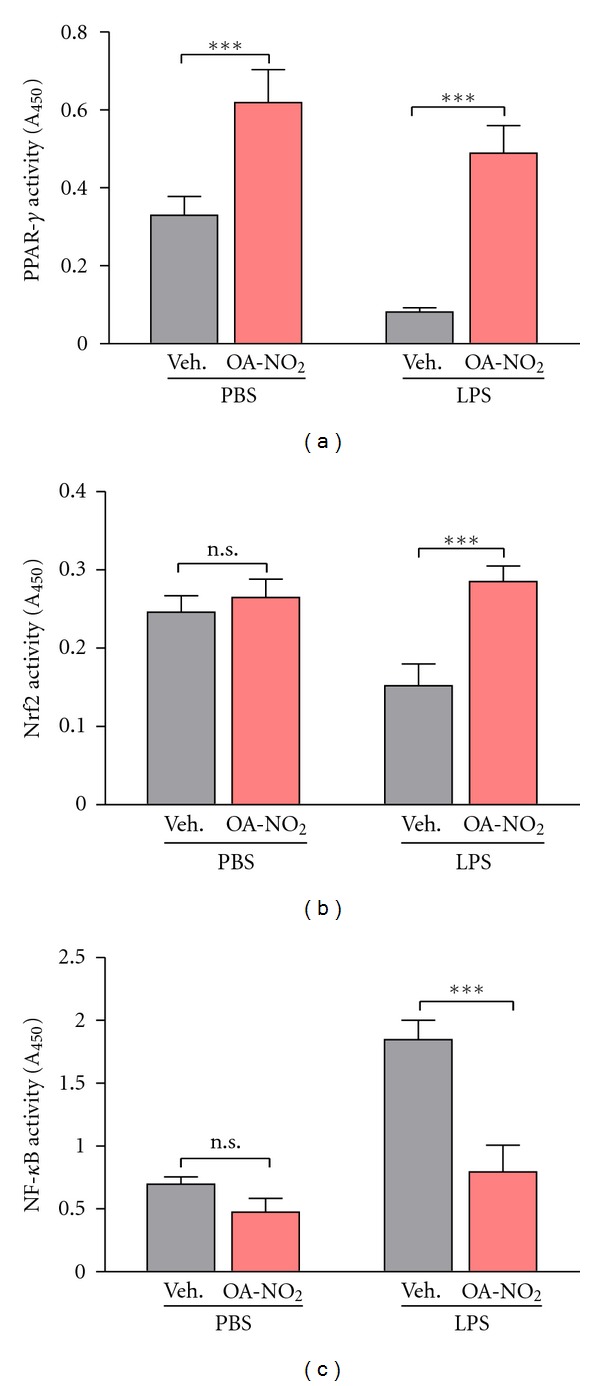
OA-NO_2_ reverses LPS-induced changes in transcription factor activity. Induction of ALI by i.t. injection of LPS (50 *μ*g) was followed 30 min later by i.t. administration (50 *μ*L) of OA-NO_2_ (50 *μ*g) or vehicle (10% DMSO). After another 5.5 h lungs were excised and DNA-binding activity of the transcription factors (a) PPAR-*γ*, (b) Nrf2, and (c) NF-*κ*B was determined. Data are representative of one of two independent experiments with *n* = 6–8 mice per group; ***P < 0.001.

We propose that NFAs exert many of their anti-inflammatory effects by activating PPAR-*γ*, which is known to decrease activity of the proinflammatory transcription factor NF-*κ*B. PPAR-*γ* promotes transcription of the antioxidant factor Nrf2, which in turn upregulates PPAR-*γ* expression in a positive feedback loop [20, 21]. To test our hypothesis, we examined the effects of OA-NO_2_ treatment on activity of PPAR-*γ*, NF-*κ*B, and Nrf2. When followed by vehicle treatment, i.t. LPS upregulated the DNA-binding activity of NF-*κ*B, as expected (Figure 4(c)) but decreased activity of PPAR-*γ* (Figure 4(a)) and Nrf2 (Figure 4(b)). OA-NO_2_ treatment, however, not only diminished the increase in NF-*κ*B activity but also increased the DNA-binding activity of PPAR-*γ* and Nrf2. Under noninflammatory conditions, OA-NO_2_ treatment increased PPAR-*γ* activity but had no significant effect on basal levels of NF-*κ*B or Nrf2 activity. Given the role of NFAs as PPAR-*γ* agonists and the known anti-inflammatory and antioxidant activities of PPAR-*γ*, many exerted through inhibition of NF-*κ*B activity and upregulation of Nrf2, these data support the concept that NFAs act in part via PPAR-*γ* activation.

### 3.6. OA-NO_2_ Decreases Inflammatory Response of Alveolar Macrophages

**Figure 5:**
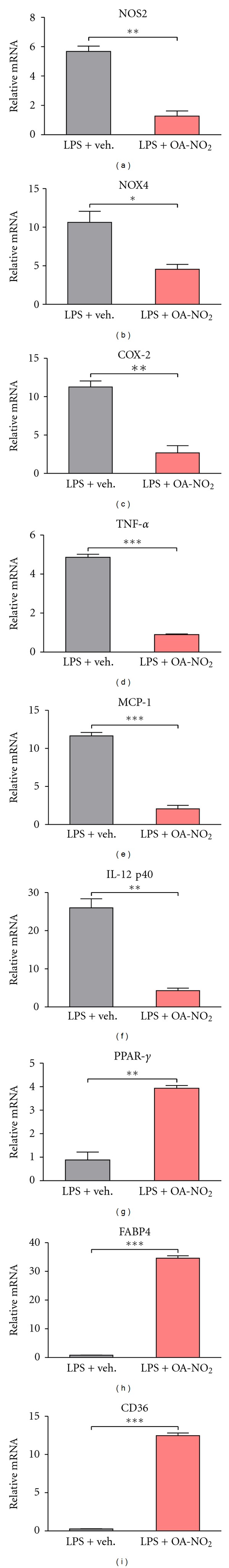
OA-NO_2_ reduces inflammatory phenotype in LPS-activated alveolar macrophages. Induction of ALI by i.t. injection of LPS (50 *μ*g) was followed 30 min later by i.t. administration (50 *μ*L) of OA-NO_2_ (50 *μ*g) or vehicle (10% DMSO). After another 5.5 h BAL fluid was obtained. Alveolar macrophages were isolated from the BAL fluid and plated in DMEM + 10% FBS. After 1 h, RNA was isolated and expression of the indicated genes was determined using real-time PCR; results were normalized to values for the housekeeping genes glyceraldehyde-3-phosphate dehydrogenase (GAPDH) and 9s rRNA. Data are representative of one of two independent experiments with *n* = 6–8 mice per group; *P < 0.05; **P < 0.01; ***P < 0.001.

Alveolar macrophages play a central role in regulation of the lung's immune system. When activated by stimuli such as LPS, they generate oxidants and secrete molecules that attract neutrophils and other immune cells to the lung. As PPAR-*γ* is known to play a major role in modulating activation of alveolar macrophages [22], we investigated the ability of NFAs to suppress LPS-induced activation of these cells. Specifically, mice were treated with LPS and either vehicle or OA-NO_2_ as previously described. Alveolar macrophages were then isolated and their RNA extracted for determination of relevant gene expression. Results (Figure 5) demonstrate significant suppression of the proinflammatory cytokines TNF-*α* and interleukin-12 (IL-12) and the chemokine MCP-1. Downregulation was also observed for the inducible form of nitric oxide synthase (iNOS; NOS2) and the superoxide-generating enzyme NADPH oxidase 4 (NOX4), both of which contribute to oxidative stress. OA-NO_2_ treatment also reduced alveolar macrophage expression of cyclooxygenase 2 (COX-2), which synthesizes proinflammatory prostaglandins. Conversely, OA-NO_2_ upregulated expression of PPAR-*γ*, and the PPAR-*γ* target genes fatty acid binding protein 4 (FABP4) and CD36, a receptor that facilitates macrophage phagocytosis of apoptotic and senescent neutrophils and thereby contributes to the resolution of inflammation. All these measurements confirm the ability of NFA treatment *in vivo* to suppress the activated, proinflammatory phenotype of alveolar macrophages.

## 4. Discussion

Our results establish that delivery of OA-NO_2_ directly to the lung significantly reduces the severity of pulmonary inflammation and injury. We also find that treatment with OA-NO_2_ suppresses the activated phenotype of alveolar macrophages, key cells in the regulation of pulmonary inflammation. In the lung, LPS-induced increase in activity of the proinflammatory transcription factor NF-*κ*B is largely blocked, as are LPS-induced decreases of PPAR-*γ* and the antioxidant transcription factor Nrf2. Indeed, PPAR-*γ* activity is increased over basal levels in both inflammatory and noninflammatory conditions. Upregulation of Nrf2 expression and activity may account for much of the antioxidant activity of PPAR-*γ* and consequent reduction in inflammation-associated oxidative injury [20, 21].

This appears to be the first study of NFAs' ability to reduce the severity of ALI and one of very few to investigate the protective anti-inflammatory actions of NFAs in an animal model of any disease. Borniquel and colleagues have recently obtained results in a murine model of inflammatory bowel disease that are similar to ours in ALI [23], finding that OA-NO_2_ decreased disease severity and the increase in NF-*κ*B expression while increasing expression of PPAR-*γ*. Significantly, the increase in PPAR-*γ* expression was abolished by simultaneous administration of a PPAR-*γ* antagonist. OA-NO_2_ was also found to reduce infarct size in cardiac ischemia-reperfusion injury [24]. This was accompanied by a reduction in NF-*κ*B activity but a potential role for PPAR-*γ* was not investigated. Beneficial effects of OA-NO_2_, together with reduced inflammation, have also been seen in renal ischemia/reperfusion injury [25]. It appears likely that a number of other inflammatory conditions might also benefit from NFA treatment.

Activation of the nuclear hormone receptor PPAR-*γ* has been shown to reduce the severity of LPS-induced ALI [5, 9], and NFAs are known to activate PPAR-*γ* [11, 12]. That this activation reflects binding to the ligand binding domain is demonstrated both by ability of NFAs to displace the synthetic ligand rosiglitazone [12] and by the elucidated crystal structure of LNO_2_ bound to this site [19]. We therefore suggest that the protective effects we observe are mediated largely by activation of PPAR-*γ*. Such activation appears adequate to account for most of our observations, including increased activity and expression of both PPAR-*γ* itself and the antioxidant transcription factor Nrf2, which both upregulates and is upregulated by PPAR-*γ* [20, 21], as well as decreased activity of the proinflammatory transcription factor NF-*κ*B.

Initial studies suggested that OA-NO_2_ and LNO_2_ were present in normal human plasma at concentrations exceeding 0.1 *μ*M, which in conjunction with their measured binding affinity would raise the possibility that they were endogenous PPAR-*γ* agonists [11]. Later studies from another group, however, have suggested concentrations below 1 nM [26]. Notably, both studies were performed with plasma from healthy subjects and do not reflect the increase in NFA concentrations anticipated in inflammatory conditions. This controversy nevertheless has only limited relevance to our studies, which address pharmacological effects of exogenously delivered OA-NO_2_ rather than the role of endogenous NFAs. 

Although the ability of  NFAs to activate PPAR-*γ* at readily achievable plasma concentrations is well established, this may not be their only mechanism of action. NFAs are known to alkylate thiol groups via the Michael reaction [27] and the large majority of plasma NFAs are in fact present as protein adducts [28]. These adducts, however, are primarily to serum albumin or other proteins unlikely to be involved in signaling pathways. Nevertheless, alkylation of signaling proteins might well underlie certain NFA effects. It has been shown that NFAs alkylate the p65 subunit of NF-*κ*B, thus reducing its DNA-binding activity [16]. This mechanism has been proposed to mediate the protective effect of NFAs on cardiac ischemia/reperfusion injury, although involvement of other mechanisms, including PPAR-*γ* activation, was not ruled out [24]. More recently it has been shown that NFA alkylation of atypical protein kinase C*ζ* inhibits bradykinin-induced Ca^++^ influx in pulmonary epithelial cells [29]. Other studies have identified NFA effects not readily related to PPAR-*γ* [30–32], including induced expression of anti-inflammatory and cytoprotective genes under control of the heat shock transcription factor [33], but these studies did not identify a specific mechanism for the observed effects. These data suggest that NFA actions involve both PPAR-*γ*-dependent and -independent mechanisms, although this question requires further investigation.

Failure of OA-NO_2_ to upregulate Nrf2 activity under noninflammatory conditions appears unexpected, given the substantial increase in PPAR-*γ* activity we observed. PPAR-*γ* knockdown has been shown to block O_2_-induced increases in Nrf2 expression [20] while PPAR-*γ* agonists upregulate activity of this antioxidant transcription factor [34]. In the latter system, however, induction was weak in the absence of additional stimulation with retinoic acid, an activating ligand for the RXR receptor with which PPAR-*γ* forms a heterodimer required for transcriptional activity. NFAs have also been reported to upregulate Nrf2 activity and expression by alkylation of the inhibitor protein Keap-1 [35–37], which both maintains a cytosolic location for Nrf2 and marks it for ubiquitination and subsequent degradation. Other evidence suggests that NFAs may activate PPAR-*γ* and Nrf2 by distinct post-transcriptional pathways, with the latter involving phosphatidylinositol-3-kinase and protein kinase C [38]. Notably, stimulation of reporter gene activity required NFA concentrations at least 10-fold higher for Nrf2 than for PPAR-*γ*. These different potencies may account for our observation that, under noninflammatory conditions, a single 50 *μ*g injection of OA-NO_2_ upregulated PPAR-*γ* but not Nrf2 activity. In this context, NFA effects on Nrf2 would appear to be an intriguing area for further investigation.

## 5. Conclusions

Our results in a murine model of ALI support the anti-inflammatory effects of NFAs that have been demonstrated *in vitro* and in a limited number of other disease models. They also show, for the first time, that direct pulmonary delivery of an NFA can have beneficial effects in lung disease. Inflammation is an important feature of many lung diseases, including asthma and chronic obstructive pulmonary disease, and synthetic PPAR-*γ*-activating thiazolidinediones have been proposed as treatments for these diseases. As diabetes therapies, however, these agents are known to be associated with adverse effects. Our results suggest that NFAs, and perhaps OA-NO_2_ specifically, might be attractive alternatives for these diseases as well as ALI.

## Figures and Tables

**Table 1:** Oligonucleotide primers employed.

Gene		Primer sequence	Tm ^°^C	Amplicon size (bp)
9s rRNA	F	5^′^-ATCCGCCAACGTCACATT-3^′^	57.6	115
	R	5^′^-CCGCCGCCATAAGGAGAAC-3^′^	64.5	
CD36	F	5^′^-CAGTCGGAGACATGCTTATTGAG-3^′^	60.7	151
	R	5^′^-TTTGCCACGTCATCTGGGTTT-3^′^	62.5	
COX-2	F	5^′^-TGTGACTGTACCCGGACTGG-3^′^	63.0	233
	R	5^'^-TGCACATTGTAAGTAGGTGGAC-3^′^	60.0	
FABP4	F	5^′^-GGGGCCAGGCTTCTATTCC-3^′^	61.8	114
	R	5^′^-GGAGCTGGGTTAGGTATGGG-3^′^	61.1	
GAPDH	F	5^′^-GGACGCATTGGTCGTCTGG-3^′^	63.0	204
	R	5^′^-TTTGCACTGGTACGTGTTGAT-3^′^	60.2	
IL-12 p40	F	5^′^-CAAGGCTGTTCACATTATCCCA-3^′^	60.3	107
	R	5^′^-CCAGTGTGGTCATGGACTTTC-3^′^	60.6	
MCP-1	F	5^′^-TTAAAAACCTGGATCGGAACCAA-3^′^	60.1	121
	R	5^′^-GCATTAGCTTCAGATTTACGGGT-3^′^	60.7	
NOS2	F	5^′^-ACATCGACCCGTCCACAGTAT-3^′^	62.7	177
	R	5^′^-CAGAGGGGTAGGCTTGTCTC-3^′^	61.0	
NOX4	F	5^′^-TGCCTGCTCATTTGGCTGT-3^′^	62.2	180
	R	5^′^-CCGGCACATAGGTAAAAGGATG-3^′^	60.8	
PPAR-*γ*	F	5^′^-CCATTCTGGCCCACCAAC-3^′^	66.5	479
	R	5^′^-CTGAAACCGACAGTACTG-3^′^	53.8	
TNF-*α*	F	5^′^-CCTGTAGCCCACGTCGTAG-3^′^	61.5	148
	R	5^′^-GGGAGTAGACAAGGTACAACCC-3^′^	61.4	
